# The Effect of Obesity and Increased Waist Circumference on the Outcome of Laparoscopic Nephrectomy

**DOI:** 10.1155/2017/3941727

**Published:** 2017-01-22

**Authors:** D. B. Hennessey, E. M. Bolton, A. Z. Thomas, R. P. Manecksha, T. H. Lynch

**Affiliations:** Department of Urological Surgery, St. James's Hospital, James's Street, Dublin 8, Ireland

## Abstract

*Introduction*. The prevalence of obesity is increasing worldwide. Obesity can be determined by body mass index (BMI); however waist circumference (WC) is a better measure of central obesity. This study evaluates the outcome of laparoscopic nephrectomy on patients with an abnormal WC.* Methods*. A WC of >88 cm for women and >102 cm for men was defined as obese. Data collected included age, gender, American Society of Anaesthesiologists (ASA) score, renal function, anaesthetic duration, surgery duration, blood loss, complications, and duration of hospital stay.* Results*. 144 patients were assessed; 73 (50.7%) of the patients had abnormal WC for their gender. There was no difference between the groups for conversion to open surgery, number of ports used, blood loss, and complications. Abnormal WC was associated with a longer median anaesthetic duration, 233 min, IQR (215–265) versus 204 min, IQR (190–210), *p* = 0.0022, and operative duration, 178 min, IQR (160–190) versus 137 min, IQR (128–162), *p* < 0.0001. Patients with an abnormal WC also had a longer inpatient stay, *p* = 0.0436.* Conclusion*. Laparoscopic nephrectomy is safe in obese patients. However, obese patients should be informed that their obesity prolongs the anaesthetic duration and duration of the surgery and is associated with a prolonged recovery.

## 1. Introduction

Obesity is the excessive accumulation of body fat to such an extent that it has a negative impact on health. An excess of 600 million adults are obese and the epidemic of obesity has become a major worldwide health concern. For urologists, managing patients with obesity has become part of everyday practice [1]. When considering surgery in such patients, it must be taken into account that obese patients are more likely to have cardiovascular disease, hypertension, and diabetes therefore increasing their anaesthetic risk. Obesity can also make open and laparoscopic surgery more difficult and is also an independent risk factor for perioperative morbidity and has been demonstrated to be a specific risk factor for complications after surgery [2–5].

Several studies have examined the impact of body mass index (BMI) as a measure of obesity on laparoscopic surgery. However, BMI measures total obesity rather than central obesity. Central obesity is a more important measure of central abdominal fat and is a better predictor of morbidity than total obesity [6]. Measuring waist circumference (WC) is a proven alternate method to define central obesity and is a superior quantifier of central obesity related morbidities than BMI [6–8]. We aim to examine the effect of central obesity as defined by abnormal WC on the outcome of patients undergoing laparoscopic renal surgery.

## 2. Methods

Data was collected prospectively on consecutive patients from January 2011 to January 2013 who underwent laparoscopic radical nephrectomy for malignant renal disease or laparoscopic simple nephrectomy for benign renal disease or a nonfunctioning kidney. All procedures were performed by a single laparoscopic surgeon. Data were recorded prospectively on each patient in a database, which was completed immediately postoperatively by the operating surgeon or assistant.

Data obtained included age, sex, American Society of Anaesthesiologists (ASA) score, renal function (baseline and postoperative) WC, surgical approach, number of ports used, method for ligation of hilum, anaesthetic duration (defined as duration from induction of anaesthesia to extubation), surgery time, complications, and duration of inpatient stay. Patients undergoing laparoscopic nephron sparing surgery were excluded. A waist circumference of >88 cm for women and >102 cm for men was used to define central obesity as per European clinical practice guidelines [9, 10]. For the purpose of this study, patients who do not reach the WC for obesity comprise Group 1, while patients who met the criteria for central obesity were classed as Group 2.

Laparoscopic nephrectomy was performed though a three-port method, 10 mm camera port, 5 mm upper port, and 12 mm lower port. 5 mm port and 12 mm port were placed 3-4 cm superior and inferior lateral to camera port. If an additional forth port was needed, a 5 mm port was placed 3 cm medial and superior to the anterior superior iliac spine (ASIS). Routine postoperative care was provided and each patient was followed up for a minimum of 1 year. Intraoperative and postoperative complications were assessed according to the modified Clavien classification [11]. Axial abdominal computerised tomography (CT) was used to determine the WC [12]. CT scans were performed on a Siemens Emotion CT (Erlangen, Germany) using a 5 mm slice thickness for acquisition and reconstruction. WC was defined at the abdominal circumference at a level midway between the lowest rib and the iliac crest. OsiriX DICOM software (Geneva, Switzerland) was used to measure the abdominal perimeter using a free-hand elliptical ROI following the skin contour, representing the use of a measuring tape.

Unless otherwise stated, data is represented as median (interquartile range: IQR) and *N* represents the number of patients included in the analysis. Differences in distribution of clinical data were evaluated using Mann-Whitney *U* test or Fishers exact test. All calculations were done using Prism version 5.0 (GraphPad Software, Inc., La Jolla, CA). A significant difference was defined as *p* < 0.05.

## 3. Results

### 3.1. Patient Characteristics

144 patients underwent laparoscopic nephrectomy, 86 (59.7%) were male, 58 (40.3%) were female. 71 (49.3%) of patients were not obese (Group 1) and 73 (50.7%) were obese (Group 2). 39 (45.5%) of male patients were obese and 34 (48.3%) of female patients were obese. Median WC of obese male patients was 108 cm, IQR (102.3–119.3), and WC of nonobese male patients was 86 cm, IQR (82.2–90). The median WC of obese female patients was 98.5 cm, IQR (94–107), and WC of nonobese females was 78 cm, IQR (75–79). Median age at the time of surgery was 60 years, IQR (52–66). 23 (15.9%) of patients were ASA grade 1, 74 (51.3%) were grade 2, 45 (31.2%) were grade 3, and 2 (1.3%) were grade 4. Group 2 patients had higher ASA grades than Group 1 patients, *p* = 0.04. There was no statistical difference in age, medical comorbidities, or preoperative renal function between Group 1 and Group 2 patients. However, Group 2 patients had more medical comorbidities than Group 1 as expected (Table 1).

### 3.2. Operative Data

**Figure 1:**
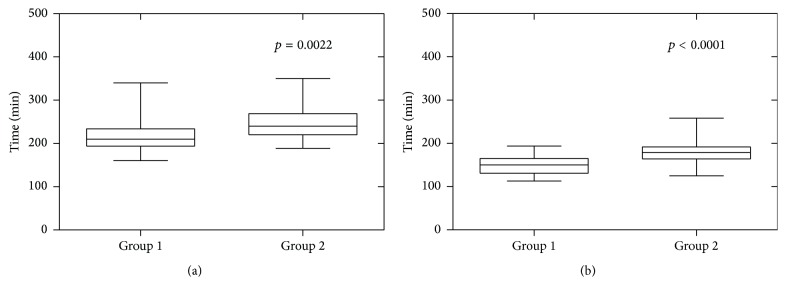
Effect of obesity on (a) anaesthetic time and (b) surgical time. (a) Effect of obesity on the duration of anaesthetic. Group 1, median 204 min, IQR (190–210) versus Group 2, median 233 min, IQR (215–265), *p* = 0.0022. (b) Effect of obesity on the duration of surgery. Group 1, median 137 min, IQR (128–162) versus Group 2, median 178 min, IQR (160–190), *p* < 0.0001.

All procedures were commenced laparoscopically with a three-port approach and 2 (1.4%) cases were converted to open surgery. There was no difference between the groups regarding conversion to open surgery. 132 (90.2%) of procedures were completed with three ports and 12 (9.8%) cases required a forth port for competition. There was a nonstatistically significant trend towards a higher use of a forth port in Group 2 patients. The renal vessels were ligated with Hem-O-lok in 140 (98.5%) cases and by endovascular automated stapler in 2 (1.5%). The median anaesthetic duration for all operations was 224 min, IQR (201–250). For Group 1 patients it was 204 min, IQR (190–210), and for Group 2 patients it was 233 min, IQR (215–265). Group 2 patients had a longer anaesthetic duration, *p* = 0.0022. The median surgical duration for all operations was 168 min, IQR (139–192). For Group 1 patients it was 137 min, IQR (128–162). For Group 2 it was 178 min, IQR (160–190) (Table 3), and Group 2 patients had a longer surgical time, *p* < 0.0001.  Figure 1 demonstrates the statistical differences in anaesthetic and surgical times between the groups. There was no difference in operative blood loss between patient groups, *p* = 0.1496, in Table 2.

### 3.3. Early Postoperative Outcomes and Complications

There was no difference in renal function at one year after nephrectomy between the groups, *p* = 0.0553. 35 patients (24.4%) had postoperative complications, 29 (74.3%) were grade I, 2 (5.1%) were grade II, 2 (5.1%) were grade IIIa, and 1 was grade IIIb. One (2.55%) patient had a grade IVa complication. There was no difference in the complication rates between patients in both groups. However, three times as many grade 3 and above complications occurred in Group 2 patients. Grade 3 complications included 2 patients who required radiological drain placement for a haematoma (1 Group 1, 1 Group 2). One patient (Group 2) developed a port site hernia and needed surgical repair and 1 patient (Group 2) was admitted to intensive care with wound haematoma, renal failure, and sepsis. Group 2 patients had a longer inpatient stay than Group 1 patients, *p* = 0.0436 (Table 3).

## 4. Discussion

Obesity is now a common problem; it is estimated that 28% of men and 29% of women in the world are obese, mainly in western societies [1, 13]. In addition to increasing rates of obesity, there is also a significant link between obesity and renal cell carcinoma (RCC) [14]. Initially, obesity was considered a relative contraindication to laparoscopic surgery [4, 15]. But, it was soon established that laparoscopic surgery in obese patients as defined by an elevated body mass index (BMI) was safe [15, 16]. However, BMI measures total obesity, by deriving a figure from the patient's height and weight. Frequently, professional athletes with significant muscle mass can be defined as obese by this method. BMI does not accurately measure central obesity, which is a more important determinant of medical comorbidity and postoperative morbidity [6]. Measuring WC directly measures central obesity and is a better predictor of central obesity related morbidities [6–8]. WC is superior to BMI in predicting the development of numerous chronic diseases such as type 2 diabetes and cardiovascular disease (CVD) and was found to be an independent risk factor for the development of complications in colorectal surgery patients [17].

This is the first study to determine the impact of WC on surgical outcomes for patients undergoing laparoscopic renal surgery. We found that patients with an abnormal WC compared to normal WC patients had statistically significant longer anaesthetic time, operative times, and hospital admission. There was no difference between obese and nonobese patients in relation to increased risk of blood loss, postoperative renal dysfunction, or complications.

Obese patients are more likely to have a longer anaesthetic duration compared to lean patients for a number of reasons. Firstly, gaining intravenous access in this patient group can be difficult [18]. Secondly, the rate of difficult tracheal intubation is much higher in this group; Juvin et al. reported a rate of 15.5% compared to 2.2% in normal patients [19]. Finally, obese patients have increased risk for adverse respiratory events secondary to anaesthetic agents. This is in part due to altered pharmacokinetic and pharmacodynamic differences in morbidly obese individuals. But it is also related to fat deposition in the pharynx and chest wall altering respiratory function [20].

The duration of operative times in obese patients was longer for three reasons. Firstly, gaining access in these individuals was challenging due to the excess adiposity. We also noted that we had to alter port placement. This was due to the pannus of the obese patient shifting disproportionately the umbilicus. As a result we had to place the trocars more laterally to reduce the distance to the area of interest. In all of our patients, laparoscopic port placement was achieved using the Hassan technique. A possible solution to difficult trocar placement in obese patients with the Hassan technique is the use an optical bladed and bladeless trocar. Bladeless optical trocars have a clear conical tip with flanges that separate fascial and muscle fibres as the trocar is pushed through the abdominal wall. The bladed trocar has a clear half-sphere dome that allows tissue visualization. Its blade is activated by a trigger mechanism and cuts the tissue in view, then automatically retracts. Bernante et al. reported that in a series of 200 consecutive laparoscopic bariatric procedures with bladed optical access trocar the average trocar insertion time was 20 seconds [21]. Similarly, Sabeti et al. reported the use of the optical trocar in over 2200 patients and found that the device was extremely safe, with only a 0.18% complication rate [22].

Secondly, the increased intra-abdominal fat of obese patients prolonged the surgical time. Increased intra-abdominal adipose tissue makes mobilisation of the bowel, identifying and isolating the ureter, renal artery, and vein, and dissection of the kidney more demanding and time consuming. In addition, we found in some cases that excessive intra-abdominal adipose tissue made placement of the excised kidney into the extraction bag difficult. While this has not been reported yet in regard to laparoscopic renal surgery, recently this finding has been reported to prolong operative times in other retroperitoneal surgeries [23].

Finally, closure of the laparoscopic port sites was more difficult in obese patients than nonobese patients. This was due to the fact that the adiposity increased the distance between skin and fascia making fascial closure tough. In each patient, the fascial defects were closed with retraction and J needle. There are multiple methods described to speed up the closure of a port site fascial defect [24]. However no one technique has found universal acceptance. Perhaps in the near future an automated laparoscopic port closure device will be introduced that will decrease port closure times [25].

The relatively small patient numbers included in this study is a limitation that we should report. It is conceivable that if there was more patients in the analysis, more differences between the groups may become apparent. In particular, the use of a forth port, blood loss, and postoperative complications may be higher in the obese group of patients. Another limitation of this study is that we measured renal function at one year after nephrectomy; it is believable that a longer follow-up might show a difference in renal function between the groups.

## 5. Conclusion

Laparoscopic renal surgery is more challenging in patients with an abnormal WC but is feasible. Patients with an abnormal WC have longer anaesthetic time and longer surgical time and take longer time to recover in hospital but have a complication profile similar to that of nonobese patients. Obese patients should be cautioned that their obesity is associated with increased difficulty at the time of surgery compared to nonobese patients.

## Figures and Tables

**Table 1:** Patient demographics.

Characteristic	Total	Group 1	Group 2	*p* value
Total	144	71 (49.3%)	73 (50.7%)	

Age (years)	60 (52–66)	60 (51–69)	58 (48–66)	0.41

Gender				
Male	86 (59.7%)	47 (66.1%)	39 (53.4%)	0.12
Female	58 (40.3%)	24 (34.9%)	34 (46.6%)	

ASA grade				
1	23 (15.9%)	18 (78.2%)	5 (21.8%)	^*∗*^0.04
2	74 (51.4%)	35 (47.2%)	39 (52.8%)	
3	45 (31.3%)	18 (40%)	27 (60%)	
4	2 (1.4%)	0 (0%)	2 (100%)	

Comorbidities				
HTN	36 (25%)	14 (19.7%)	22 (30.1%)	^#^0.17
Diabetes	7 (4.8%)	4 (5.6%)	3 (4.1%)	0.71
Renal disease	4 (2.7%)	1 (1.4%)	3 (4.1%)	0.62

Preoperative renal function				
Creatinine (mg/dl)	82 (66–93)	76 (57–91.75)	85 (70–94)	0.11
eGFR (ml/min)	87 (62–90)	90 (74–90)	74 (61.5–90)	0.23

*N*: number of patients. IQR: interquartile range. WC: waist circumference. ASA: American Society of Anaesthesiologists. ^*∗*^All assessed with ANOVA test. ^#^All assessed with Fishers exact test.

**Table 2:** Operative data.

Characteristic	Total *N*(%)	Group 1	Group 2	*p* value
Approach and completion				
Laparoscopic	142 (98.6%)	70 (49.6%)	72 (50.4%)	^*∗*^0.5
Converted to open	2 (1.4%)	1 (50%)	1 (50%)	

No. of ports				
3	132 (90.2%)	68 (51.5%)	64 (48.5%)	^*∗*^0.12
4	12 (9.8%)	3 (25%)	9 (75%)	

Ligation of hilum				
Hem-O-lok	140 (97.2%)	70 (50%)	70 (50%)	^*∗*^0.49
Endoscopic stapler	2 (1.4%)	0 (0%)	2 (100%)	
Suture	2 (1.4%)	1 (50%)	1 (50%)	

Anaesthetic time (min, IRQ)	224 (201–250)	204 (190–210)	233 (215–265)	^#^0.0022

Surgery time (min)	168 (139–192)	137 (128–162)	178 (160–190)	^#^<0.0001

Blood loss (ml)	140 (50–205)	130 (50–150)	150 (50–300)	^#^0.1496

^*∗*^All assessed with Fishers exact test. ^#^Assessed with Mann-Whitney *U* test. *N*: number of patients. No. of ports: number of ports. WC: waist circumference. GIA: gastrointestinal anastomosis. min: minutes. ml: millilitre.

**Table 3:** Postoperative outcomes and complications.

Characteristic	Total	Group 1	Group 2	^*∗*^ *p* value
Postoperative renal function				
Creatinine (mg/dl)	107 (85–133)	100 (80.2–125.5)	119 (90–145)	^#^0.0553
eGFR (ml/min)	59 (48–70)	60 (56–76)	57 (43–69)	^#^0.3446

Complication				
No	109 (75.6%)	57 (52.2%)	52 (47.8%)	^*∗*^0.206
Yes	35 (24.4%)	14 (35.8%)	21 (64.2%)	

Grade I	29 (74.3%)	12 (41.3%)	17 (48.7%)	
Grade II	2 (5.1%)	1 (50%)	1 (50%)	
Grade IIIa	2 (5.1%)	1 (50%)	1 (50%)	
Grade IIIb	1 (2.55%)	0	1 (100%)	
Grade IVa	1 (2.55%)	0	1 (100%)	
Grade IVb	0	0	0	
Grade V	0	0	0	

Inpatient stay (days)	5 (4–6)	5 (4-5)	6 (5–7)	^#^0.0436

^#^Assessed with Mann-Whitney *U*.  ^*∗*^All assessed with Fishers exact test. *N*: number of patients. WC: waist circumference. eGFR: estimated glomerular function rate.
